# Occurrence of Anthropogenic Debris in Three Commercial Shrimp Species from South-Western Ionian Sea

**DOI:** 10.3390/biology11111616

**Published:** 2022-11-04

**Authors:** Claudio D’Iglio, Dario Di Fresco, Nunziacarla Spanò, Marco Albano, Giuseppe Panarello, Federica Laface, Caterina Faggio, Gioele Capillo, Serena Savoca

**Affiliations:** 1 Department of Chemical, Biological, Pharmaceutical and Environmental Sciences, University of Messina, Viale F. Stagno d’Alcontres 31, 98166 Messina, Italy; 2 Institute for Marine Biological Resources and Biotechnology (IRBIM), National Research Council (CNR), Section of Messina, Spianata San Raineri 86, 98122 Messina, Italy; 3 Department of Biomedical, Dental and Morphological and Functional Imaging, University of Messina, Via Consolare Valeria 1, 98125 Messina, Italy; 4 Department of Veterinary Sciences, University of Messina, 98168 Messina, Italy

**Keywords:** marine pollution, Decapoda, low density microplastics, deep-sea, Mediterranean Sea

## Abstract

**Simple Summary:**

Plastic litter is ubiquitous in the marine environment due to its rapid dispersion and great durability. Furthermore, several environmental processes can modify the characteristics of plastics, altering their density and, consequently, their likelihood of sinking. In fact, deep-sea environments are highly threatened by plastic waste, with a greater risk for benthic species. The Ionian Sea is heavily impacted by man-made floating debris, accumulated on beaches or on the seabed. The aim of this work was to evaluate the presence of anthropogenic debris in the gastrointestinal tracts of three decapods (*Parapenaeus longirostris*, *Aristeus antennatus*, *Aristaeomorpha foliacea*) from the southwestern Ionian Sea. A total of 230 anthropogenic debris were isolated from 136 specimens, with a high frequency of occurrence in all analyzed species (76% in *P. longirostris*, 70% in *A. antennatus* and 83% in *A. foliacea*) mainly represented by fibers (92.6%) with a size between 0.10 and 0.49 mm, and with a predominance of blue color. The results of this study, highlight the importance of expanding knowledge on these Decapoda species of high commercial and ecological value, in a heavily impacted basin, such as the Sea Mediterranean, helping to monitor possible risks to human health.

**Abstract:**

Deep Sea environments represent the final collector of anthropogenic debris mainly represented by both plastic and non-plastic materials with different size. This led to potential contamination of deep marine fauna due to direct and indirect ingestion, representing a potential hazard for the species itself and for the final consumer. In this framework, the present study explored the occurrence of anthropogenic debris in the gastrointestinal tract of three Decapoda species of high commercial and ecological value (*Parapenaeus longirostris*, *Aristeus antennatus*, and *Aristaeomorpha foliacea*) from south-western Ionian Sea. After morphometrical measurements and sex determination, the gastrointestinal tract of 136 specimens were extracted and then chemically digested. A total of 230 low density microparticles were isolated, with a high frequency of occurrence in all the analyzed species (76% in *P. longirostris*, 70% in *A. antennatus*, and 83% in *A. foliacea*) mainly represented by fibers (92.6%) with a size between 0.10 and 0.49 mm, and with a dominance of the blue color. The results of the present study report for the first time the anthropogenic debris presence in the studied Decapoda from south-western Ionian Sea, highlighting the necessity to broaden the knowledge about anthropogenic debris pollution status in Mediterranean deep-sea species.

## 1. Introduction

The massive production of plastics materials, and their accumulation in the environment due to insufficient recycling practices have led the scientific community and the entire society to focus the attention on the risks associated with plastic contamination, both for ecosystems and human health [1]. Concerning the marine environments, these are hardly threatened by plastic litter so far as to induce the control authorities on food (e.g., EFSA, European Food Safety Authority) to establish monitoring on plastic contamination especially for seafood products [2,3]. Several studies [4,5] have highlighted how in 2010 between 4.8 and 12.7 million tonnes (Mt) of plastics entered the oceans, drawing the attention toward the increasing trend of plastic input into the environment which could reach 12,000 Mt by 2050.

Both terrestrial and maritime human activities are responsible for the continuous release of plastic into the marine environment. Once released into the sea, microplastics can colonize all compartments of the marine environment: coasts, water surface, water column, seabed, and biota [6,7]. These contaminants are considered ubiquitous due to their rapidity in dispersion related to positive buoyancy (plastics materials have low densities) and great durability [8]. Indeed, it has been observed that plastic accumulations on the sea surface represent only about 1% of the estimated global budget, while most of the remaining 99% of marine plastic will sink to the deep sea [9] due to of vertical transport from surface accumulation. However, it has recently been shown that the spatial distribution and final fate of microplastics are strongly controlled by bottom currents [9]. Microplastic transport is a difficult topic as transport includes physical, chemical, and biological processes [6]. Among the various difficulties, it should also be considered that the physical properties (e.g., size, shape, density, buoyancy) of microplastics can vary considerably, influencing their transport [10,11,12]. Their final destination seems to be mainly influenced by the density of the polymers: polymers with a density higher than that of water (>1.027 g/cm^3^) will tend to settle on the bottom; while low density polymers will tend to float on the water column [6,13]. However, the presence of low-density polymers was also found at a depth of 10,000 m [14] contradicting this hypothesis. Alternative hypotheses suggest that other factors, such as biofouling, also contribute to modifying the density of microplastics and consequently their expected distribution in the water column.

Furthermore, other processes such degradation and fragmentation processes can modify the density of microplastics and consequently their distribution in the marine environment. The distribution of microplastics, mainly the floating ones, is also influenced by environmental factors, such as winds, surface currents, turbulent flows, tides, waves, storm surges, through horizontal and vertical transport [10]. Different hydrodynamic processes, such as currents, tides, waves are the main agents of horizontal dispersion of microplastics from their sources. Microplastics, particularly floating ones, are passively transported by complex physical flows, resulting in a wide variability in surface concentrations. Wind also affects the distribution of floating plastic [15,16]. Neutral microplastics can float on the surface of the water but are also suspended in the water column until they reach deep water. Several studies have highlighted a discrepancy between the observed and predicted plastic concentrations in surface waters [17,18], also obtaining very different and more or less homogeneous vertical dispersion results depending on the oceanographic characteristics of the investigated study. This observed variability has promoted research on the vertical distribution of microplastics in the water column, leading to the evaluation of all environmental factors or intrinsic properties of plastic particles that can influence their vertical transport and subsequent sinking.

This phenomenon is well documented by the high presence of plastics and other anthropogenetic debris in deep environments and sediments, with an increased risk for species strictly related with sea floor, and meso-bathy pelagic environments [19,20,21].

Moreover, the fragmentation processes, which induce the formation of small fragments and fibers from plastics macro litter, increase their dispersion and bioavailability for marine organisms [22]. Microplastics (plastic’ fragments smaller than 5 mm [23]) are widespread distributed and ingested by marine organisms inhabiting all the domains [22,24,25,26,27], but until now their effects on organisms are less known, despite the increasing amount of experimental studies focusing on this topic. In addition to plastics, other anthropogenic debris (e.g., rayon, dyed cotton fibers) are widely distributed in the entire marine ecosystem, raising major concerns about their toxicity, bio availability, and persistence in the environment [28]. Indeed, despite it is well-known that microplastics have the capability of absorb chemical contaminants, increasing the pollutants availability for organisms due to plastics ingestion, the knowledge base on contaminants transports conveyed by other anthropogenic debris, especially natural, or semi-natural fibers, is limited, if compared with those on plastics [28,29].

The Mediterranean Sea represents one of the most polluted area in terms of anthropogenic debris in the world [30,31], with a great amounts of surface plastic and microplastics [32,33] related to the high urbanization of the coastlines and the presence of heavily polluted rivers, which act as waste source for the entire basins. Each year, 0.57 million tons of plastic enter the Mediterranean waters, and this number will continue to rise as plastic waste production is expected to quadruple by 2050 [34]. Anthropogenic debris contamination, together with the other anthropogenic impacts acting in the Mediterranean Sea, makes this semi enclose basin a hotspot for habitat degradation and environmental pollution. For this reason, it is essential to monitor and study the level of pollution and contamination of the Mediterranean Sea [35], especially in the most impacted and anthropized geographical areas [36]. The Ionian Sea is a considerably exploited area by a large trawling fleet and a developed fishery operating with different gears (longline, gillnet, purse seine). This basin is characterized by the presence of heavily impacted zones by anthropogenic debris, floating, accumulated on the beaches or on the sea bed [37,38,39]. Moreover, it is well documented the widespread presence of marine debris and fishing litter in deep benthic environment and sediment from the entire Mediterranean basin at different depths, with accumulation zones reported in many different areas (e.g., French Mediterranean coast, Tyrrhenian Sea, Eastern Mediterranean, Cilician Coast, Spanish continental shelf, Sardinian coast) [39,40,41,42,43,44,45], making essential to assess the occurrence of anthropogenic debris in deep benthic species. In this regard, it is now known that vagile benthic fauna is particularly exposed to the risk of MPs ingestion. The feeding behavior of some species of crustaceans allows them to interact with sediment-water flows and resuspended sediments, making them excellent candidates for the role of bioindicators of MPs contamination of the seabed [46,47]. This has raised several concerns, considering that some decapods species, represent an essential resource for commercial fisheries, being among the most valuable and appreciated sea food resources worldwide [48,49,50,51]. In addition to their commercial value, they play a fundamental ecological role in benthic ecosystem, being an important component of megafaunal assemblages, occupying an high trophic position, and being among the most essential preys’ for many apical demersal predators [27,51,52,53,54,55,56,57,58].

In this context, the aim of the present paper was to evaluate the presence of anthropogenic debris in the gastrointestinal tracts of three decapods of high commercial value (*Parapenaeus longirostris*, H. Lucas, 1846, *Aristeus antennatus*, Risso, 1816, *Aristaeomorpha foliacea,* Risso, 1827) from south-western Ionian Sea. They are usually caught using trawling nets, according with their bathymetric distribution. They inhabit the deep benthic environment, with the highest density at depths ranging from 150 to 350 m for *P. longirostris,* 300 to 2000 m for *A. antennatus,* and 300 to 800 m for *A. foliacea.* Several studies were carried out on microplastic contamination in *P. longirostris* and *A. antennatus* from different geographical Mediterranean areas [46,59,60,61], while only one report of plastic ingestion exists regarding *A. foliacea* [62]. Evaluating and analyzing the contamination in these species is essential to assess both the possible risk for human health related to their consumption, and the pollution degree of the deep-sea benthic environment in the studied area.

## 2. Materials and Methods

### 2.1. Sampling Area and Samples Processing

A total of 136 specimens (50 *P. longirostris*, 50 *A. antennatus*, 36 *A. foliacea*), were obtained from the local market, caught in the south-western Ionian Sea (autumn–winter 2021) by the trawling fleets operating in the Sicilian Ionian coast. This is an oligotrophic basin characterized by a high anthropogenetic impact [63,64], with a significant fishing pressure on the stocks inhabiting this area. Once landed, collected frozen specimens were transported to the laboratory to be processed. Each individual was weighted (total weight, TW) and measured (carapace length, CL), evaluating also its sex and degree of sexual maturity, according to Follesa, M. C., and Carbonara, P. [65]. Once registered the biometrics measurements, the gastrointestinal tract of each specimen was extracted for the anthropogenic debris extraction.

### 2.2. Anthropogenic Debris Extraction Protocol

For anthropogenic debris extraction, chemical digestion of the intestines and stomachs was performed, adopting a modified version of the protocol designed by Savoca et al. [66]. Each intestine was placed in a 250 mL conical glass flask. A calculated quantity of 10% KOH solution (minimum ratio 1:5 *w*/*v*) was added to the flask, subsequently covering with aluminum foil to avoid sample contamination. To remove the organic matter, the flasks were placed in an oscillation incubator to be continuously stirred at 50 °C for 48 h. Each sample was then put into a graduated glass cylinder and hypersaline NaCl solution (15%) was added to separate the two phases by density. This procedure allows low density microdebris to float in the aqueous phase [67]. After that, the supernatant was collected and filtered through a glass fiber membrane having 0.7 µm pore size and 47 mm diameter (Whatman GF/F, UK) using a vacuum system (Millipore). Neat filters were used as blank, following the same procedure of the samples. The filters were placed in sterile glass Petri dishes for subsequent observations under the stereomicroscope to isolate the anthropogenic debris. The isolated samples were recorded and categorized based on their shape, size classes, and color. The origin of the isolated microparticles was verified using the hot needle test to observe the melting points [22]. The hot needle test is now an accepted, inexpensive method that allows to check for the presence of plastic particles based on their response; in fact, the temperature range at which melting occurs does provide a specific range of potential plastics [68]. Briefly, the tip of a fine needle was heated and each isolated microparticle was tested under a stereomicroscope. When the microparticles dissolved after exposure to the hot needle, they were confirmed as microplastics (MPs).

### 2.3. Contamination Prevention

The samples were processed in a restricted access room to prevent any accidental external contamination. Workspaces and tools were thoroughly cleaned according to [66]. During the dissection procedure the specimens were exposed to the air for the minimum time possible within a glass Petri dish. All the materials used for dissection and analysis were rigorously cleaned with ethanol and filtered deionized water. Additionally, deionized water, potassium peroxide, and hypersaline solution were always pre-filtered (0.45 mm filter). Only sterilized glass items were used for all the assays. All sample processing was performed in a clean air flow cabinet to exclude the external contamination from fibers, which might represent a major contamination source. Filter paper in Petri dishes exposed to the laboratory air was used as control blank during the analysis [69]. Procedural blanks were obtained using filtered potassium peroxide and hypersaline solution, running through the entire laboratory procedure.

### 2.4. Data Analysis

After excluding non-plastic particles, the abundance and size of isolated anthropogenic debris (ADs) have been compared between male and female specimens within the same species and among species by applying the one-way analysis of variance (ANOVA). Relations between specimens’ body weight and total length and microplastic number or size were tested using the Pearson’s correlation. The Chi-square test was used to compare the colors of ADs ingested by species. Significance level was set at *p* < 0.05. Statistical analyses were performed using the software package Prism, Version 8.2.1 (Graphpad Software Ldt., La Jolla, CA 92037, USA).

## 3. Results

In the present study, three major commercial shrimp species *P. longirostris*, *A. antennatus,* and *A. foliacea* were investigated for their content of anthropogenic debris (AD) in the gastrointestinal tract (GIT). The number of specimens analyzed and their morphological characteristics, including the total body length (TL, cm), body weight (W, g) of the analyzed species are reported as means ± SD in Table 1. Morphological characteristics of the specimens that did not show AD contamination are shown in Table 2. The size classes of the identified MPs are shown in Table 3.

A total of 136 specimens were examined. The non-plastic particles identified were excluded from the statistical analysis and were represented by 9, 5, and 11 microparticles isolated from *P. longirostris*, *A. antennatus,* and *A. foliacea*, respectively. 

**Figure 1:**
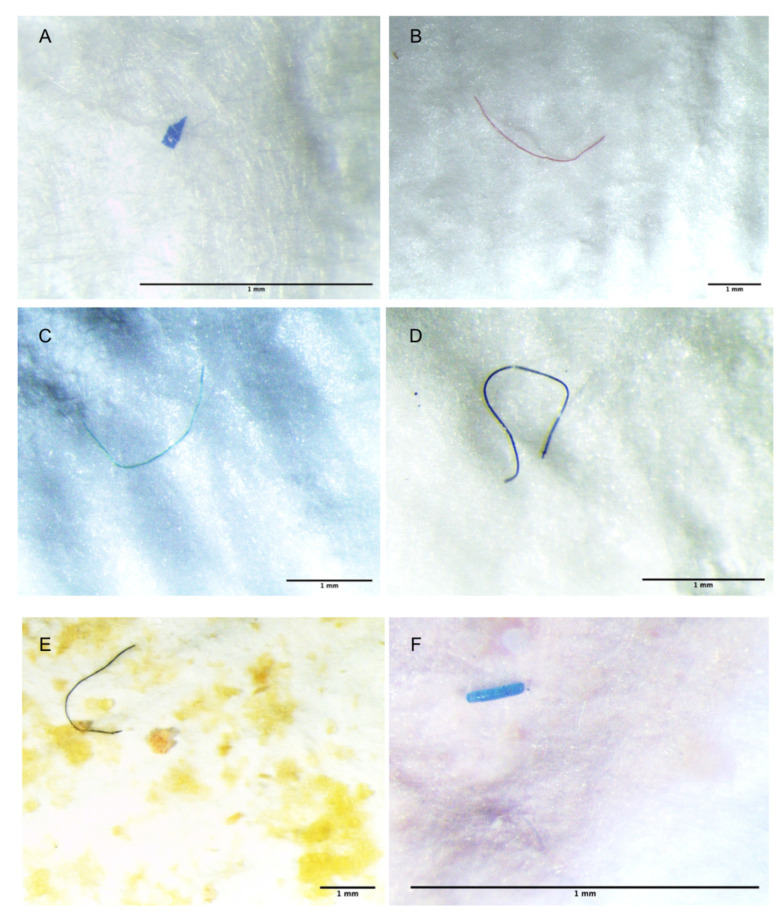
Representative images of AD isolated from *P. longirostris* (**A**,**B**), *A. antennatus* (**C**,**D**), and *A. foliacea* (**E**,**F**).

Overall, 230 MPs were isolated, mostly represented by fibers (92.6%) with a size between 0.1 and 0.49 mm (20.43%), and with a dominance of the blue color (42.6%). Representative images of the isolated MPs are shown in Figure 1. A detailed description of the results obtained for each species is reported below.

**Figure 2:**
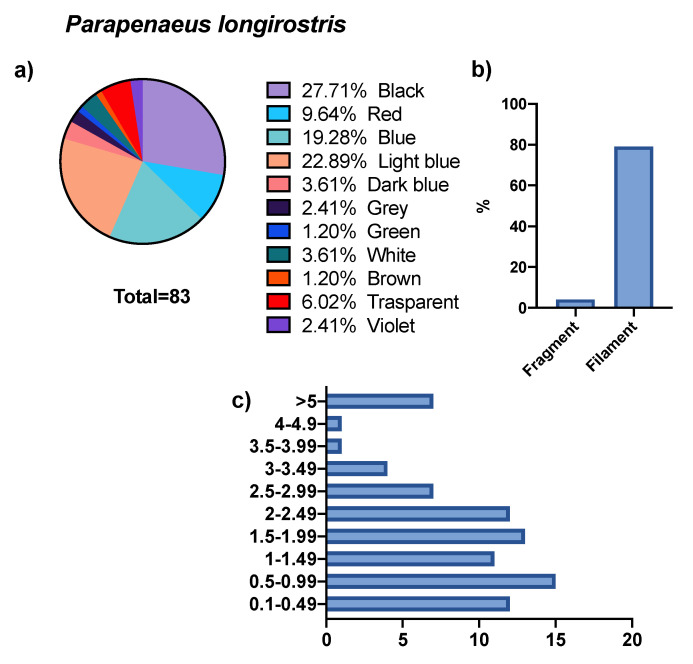
Abundance, colors (**a**), shape (**b**), and size (**c**) of microparticles isolated from *P. longirostris* specimens.

The GITs of 50 specimens belonging to the *P. longirostris* species were examined, in which the presence of MPs was found in 76% of the specimens analyzed. From these, 83 micro debris were isolated, present both in the form of fibers (95.1%) and fragments (4.8%). The size of these microparticles was between 0.11 and 10.40 mm, the largest percentage of which fell in size class II (18%). The color composition of the microparticles was rather heterogeneous, black (27.70%) and light blue (22.89%) were the dominant ones, followed by lower representative percentages of blue (19.27%), red (9.60%) and others (see Figure 2).

No difference in AD abundance was found between male and female specimens (*p* > 0.05).

**Figure 3:**
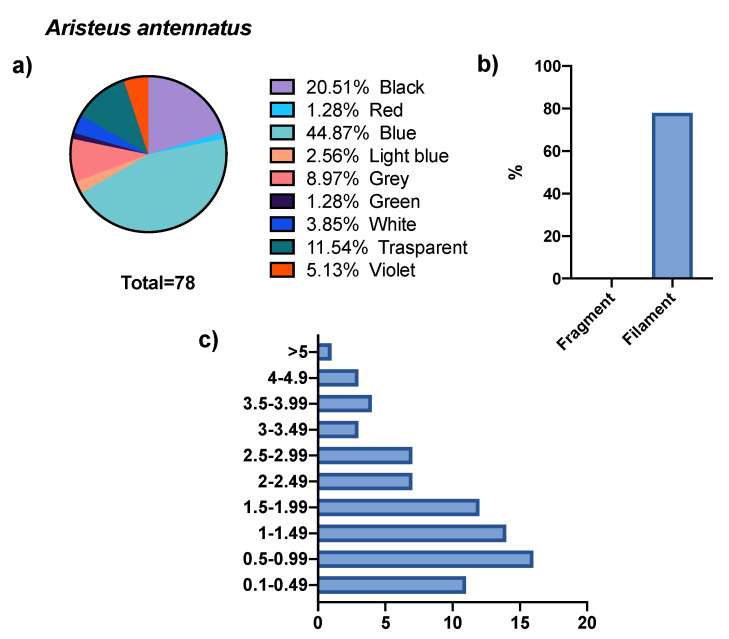
Abundance, colors (**a**), shape (**b**), and size (**c**) of microparticles isolated from *A. antennatus*.

The GITs of 50 specimens belonging to the *A. antennatus* species were examined, of which 70% showed the presence of MPs. From these, 78 microdebris were isolated, present only in the form of fibers. The size of these microparticles was between 0.11 and 5.50 mm, the largest percentage of which fell in size class II (20.5%). The color distribution of the microparticles was more characterized by the dominance of blue (44.8%) and black (20.5%), followed by lower representative percentages of transparent (11.5%), gray (8.9%), and others (see Figure 3). All the specimens were females, so it was not possible to differentiate between the sexes.

**Figure 4:**
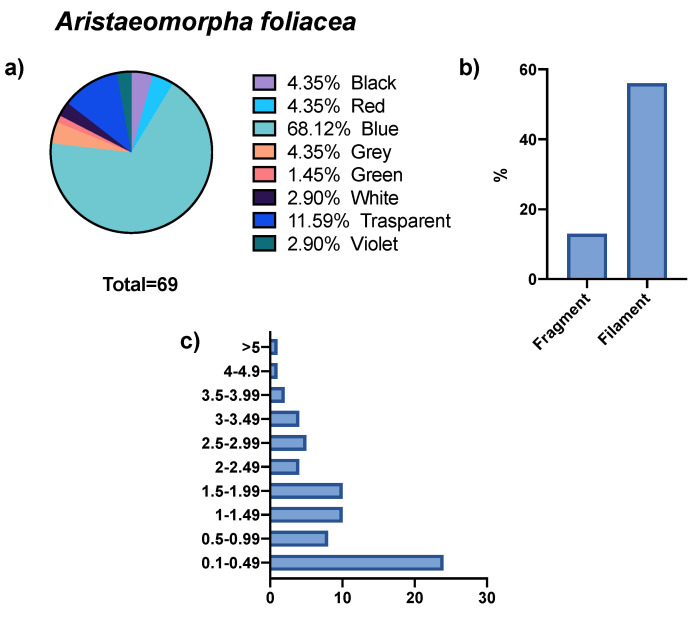
Abundance, colors (**a**), shape (**b**), and size (**c**) of microparticles isolated from *A. foliacea*.

Finally, 36 GITs of *A. foliacea* were examined, showing the presence of MPs in 83% of the specimens analyzed. From these, a total of 69 microdebris were isolated, of which 81% had a fibrous form and 18.8% a fragment form. The size of these microparticles was between 0.01 and 7.50 mm, the largest percentage of which fell in size class I (34.7%). Blue colored microparticles were dominant (68.0%), followed by transparent ones (11.6%) (see Figure 4). All the specimens were females except two, so it was not possible to differentiate between the sexes, as the result would have been inaccurate.

**Figure 5:**
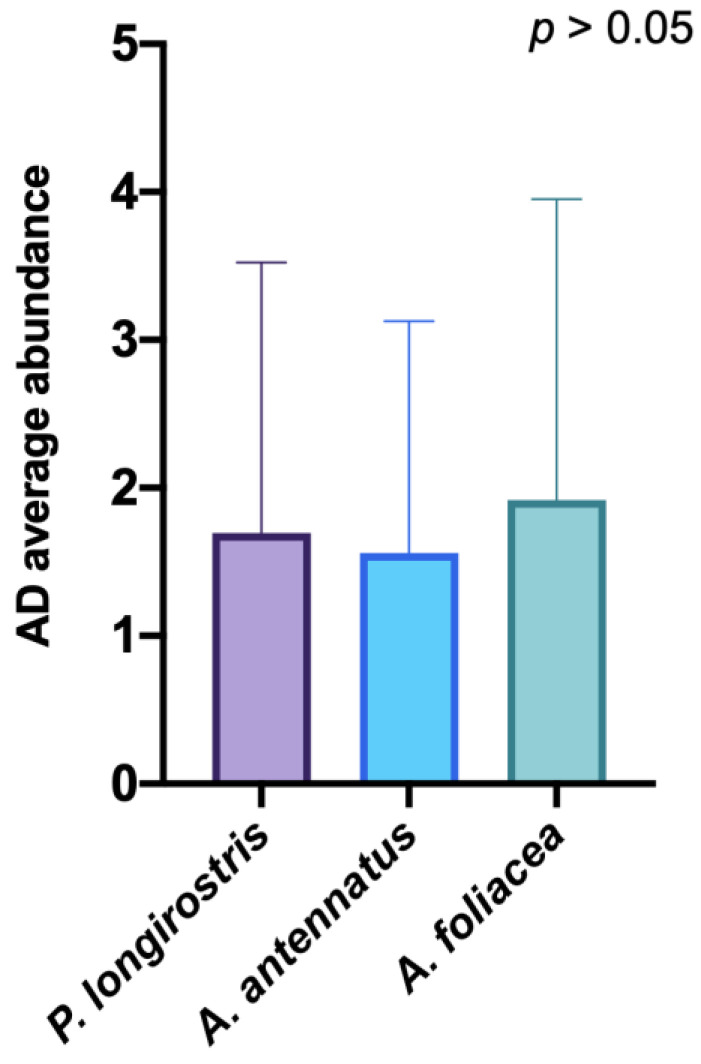
Microparticles abundance comparison between the three species analyzed.

No significant differences between the MPs abundances were found between the species. Furthermore, there was no correlation between the size of the specimens of each species and the dimensional characteristics and abundances of the MPs (*p* > 0.05, Figure 5).

**Figure 6:**
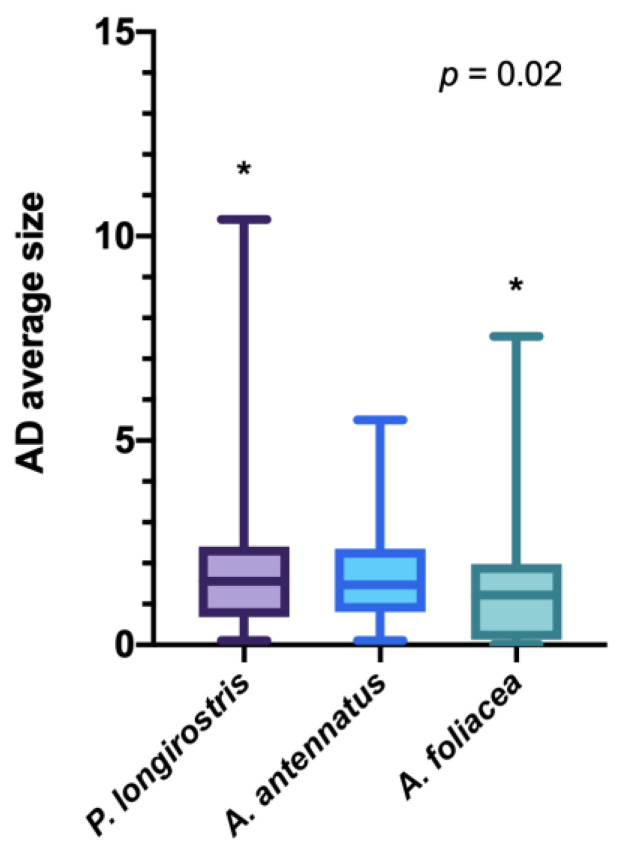
Microparticles size comparison between the three species analyzed. * indicates significant differences *p* < 0.05.

However, significant AD size differences were found between *P. longirostris* and *A. foliacea* specimens (*p* = 0.02) (Figure 6). Indeed, larger MPs were found in the first species than in the second, as shown in Table 3.

Significant differences were identified in the color composition of the MPs isolated from the three species (*p* < 0.05).

## 4. Discussion

To our best knowledge, the present paper was the first investigation on the AD presence in gastrointestinal tract of *P. longirostris, A. antennatus,* and *A. foliacea* from south-western Ionian Sea. Results showed a high frequency of debris’ occurrence in all the analyzed species (respectively 76%, 70% and 83%), which, if compared with the literature from heavily contaminated areas [70], confirm the high and worrying degree of anthropogenic debris contamination in Mediterranean Sea deep environment. Indeed, this is considered a contamination hotspot for AD (especially micro and macro plastics) both in water column, on seafloor, and in sediments [33,41,45,71,72,73,74]. Concerning the south-western Ionian Sea, as widely reported in many Mediterranean geographical sub areas, the presence of submarine canyons [42,45,75], together with the peculiar water mass circulation [76,77,78,79,80], the presence of high urbanization degree near the coast, and the large amount of fisheries activities [49,76] could increase the accumulation of debris, especially fishing gear and waste of various nature which settle on sea floor [41,43]. The fragmentation and degradation processes acting on these debris induce the formation of small fragments and microfibers (such as microplastics), enhancing their availability for benthic organisms, which accidentally (through gills [81]) or intentionally may ingest them. According to the literature, it is widely reported how marine organisms can mistake small AD for food [82], ingesting them by a direct way or via indirect intake through trophic transfer [83].

Concerning investigated species, these are active benthic predators, with secondary scavenging habits [55,84,85]. *P. longirostris* alternate a hunting phase, in which it preys on swimming benthopelagic species (e.g., crustaceans, cephalopods and small fishes), with a digging phase, in which it digs in the mud searching for food, such as polychaetae, echinoderms, and bivalves [85]. It is widely distributed in depth not exploited by *A. antennatus* and *A. foliacea*, showing a different bathymetrical distribution (from 50 to 700 m, with highest densities in Mediterranean Sea reported between 150 to 350 m), fundamental for a resource partitioning with the other bathyal penaeoideans [51,86]. This difference in distribution was highlighted also by the color of micro debris isolated from analyzed specimens, with a dominance of black (27.70%) and light blue (22.89%) fibers, bigger than those found from the other species. The color and size composition of anthropogenic debris isolated from *P. longirostris* specimens could be strictly related to bathymetry and habitats exploited by the species. Indeed, *A. antennatus* and *A. foliacea,* inhabiting deeper environments than *P. longirostris*, showed a similar dimensional range (0.11–5.50 mm and 0.10–7.50 mm, respectively) with a closer color composition (blue 44.8% and black 20.5%, blue 68.0%, respectively) of micro debris isolated from GIT. According to previous literature on AD contamination in *P. longirostris*, only one study was performed on specimens from the Strait of Sicily [60]. Results obtained by Bono et al. [60] had been very different from those obtained in the present paper, with a lower frequency of occurrence (21%), the presence of spherical fragments, and a relation between plastic occurrence and shrimps’ size. These differences could be related to the different sampling area, highlighting the high contamination degree of south-western Ionian Sea deep environments. Concerning the relation between debris occurrence and shrimps’ length, further analysis with a larger dimensional range of samples is required to analyze the potential connection between length and debris contamination. As reported by several authors, *P. longirostris* diets show ontogenetic variation, with large specimens which show the most efficiency as active predators than smaller ones [85,87]. This variation in predation dynamics could also influence the anthropogenic debris intake, facilitated or not by the increase in active predation.

As stated before, *A. antennatus* and *A. foliacea* showed a similar composition for fibers color and size, with a difference in micro debris shape. All the AD isolated from *A. antennatus* samples were fibers, while *A. foliacea* samples showed the highest occurrence of fragments (18.8%) among the studied species. This may be related to their different feeding habits. Indeed, as widely reported in the literature, these two sympatric species have been adapting to exploit different resources to facilitate their coexistence in similar areas [88]. *A. antennatus* is an euryphagous species adapted to hunt endobenthic invertebrates in the mud [89,90]. Otherwise, *A. foliacea* diet is mainly based on planktonic and pelagic species (e.g., euphausiids, myctophids) [55,91,92]. These different feeding habits could influence the intake dynamics of plastics and other AD, allowing the differences in debris shape showed by results. The AD contamination in *A. antennatus* GIT was previously assessed in the literature from other Mediterranean geographical area. Carreras-Colom E. [59,61,93] analyzed the contamination with microplastics in this species from the Balearic Basin (northwestern Mediterranean Sea), investigating also the seasonal and geographical dynamics in plastics occurrence and their impact on shrimps health condition. The frequency of occurrence in 2020 [59] was higher (85.8%) than that reported in results from the present paper, with the massive presence of single fibers and tangled ball of fibers. This high degree of plastic contamination in GIT of *A. antennatus* from high impacted Mediterranean geographical areas, such as Balearic Basin area near Barcelona city, confirms once again the importance of monitoring the contamination of anthropogenic debris in deep benthic organism, and how this can be strictly related to the degree of environmental pollution.

Concerning *A. foliacea,* to our best knowledge, the present paper represents the first assessment on the presence of AD in GIT, since, according to the literature [55], only one study on diet and trophic ecology had reported the presence of plastic debris in stomach contents of samples from Western Mediterranean Sea. The high frequency of occurrence showed by results (83%), with the dominance of blue fibers isolated from samples, underlines the necessity to improve the knowledge base on the presence of plastics and other AD in GIT of deep benthic crustaceans, especially of those with high commercial value. Indeed, despite it is widely reported in the contamination in many animal species inhabiting marine environments [82,94], relative less studies have been performed worldwide on shrimps and other decapod crustacean species despite their high commercial and ecological value [70]. For this reason, it is essential to broaden the knowledge base on this essential invertebrate class in a highly impacted basin, such as the Mediterranean Sea, focusing the attention on the most commercially viable species, and also monitoring the possible risks for human health.

## 5. Conclusions

The present study assessed the presence of anthropogenic debris in the gastrointestinal tracts of the studied species, *P. longirostris*, *A. antennatus,* and *A. foliacea*. A total of 230 low density microparticles were isolated, with a high frequency of occurrence in all the analyzed species (76% in *P. longirostris*, 70% in *A. antennatus*, and 83% in *A. foliacea*) mainly represented by fibers (92.6%) with a size between 0.10 and 0.49 mm, and with a dominance of the blue color. To our best knowledge the results obtained in this study report for the first time the anthropogenic debris presence in the studied Decapoda from south-western Ionian Sea, highlighting the necessity to broaden the knowledge about anthropogenic debris pollution status in Mediterranean deep-sea species. This could help also to monitor possible risks of ingestion in humans, only in case of consumption of the individual’s whole body (without evisceration). Additionally, it will be of fundamental importance to perform studies on the potential presence of nano-sized debris in edible tissues to better assess the risks of these pollutants’ ingestion.

## Figures and Tables

**Table 1:** Morphometric data of the analyzed crustacean species collected from the south-western Ionian Sea and the corresponding levels of particle contamination. N: number of specimens examined; Np: number of samples with detected particles.

Species	Length (mm)	Weight (g)	N° of Specimens	Np	Items/Specimen
	Means ± SD	Means ± SD			
*Parapenaues longirostris*	23.3 ± 1.6	8.1 ± 1.5	50	37	2.24
*Aristeus antennatus*	45.8 ± 5	31.5 ± 8	50	35	2.22
*Aristaeomorpha foliacea*	38.4 ± 6.7	19.1 ± 11	36	30	2.30

**Table 2:** Morphometric data of the analyzed crustacean species collected from the south-western Ionian Sea that did not show anthropogenic particles contamination. Maturity stages (see Maturity column) were detected according to the Atlas of the maturity stages of Mediterranean fishery resources [65]; 2E represents the resting adults stage in female specimens (uncolored resting ovaries with the presence of spermatophores in *A. antennatus* and *A. foliacea*) and 2B represents the recovering stage in both female (ovary developing status with a flesh, ivory and cream color in *A. foliace, A. antennatus* and *P. longirostris,* respectively) and male specimens (petasma completely joined, without spermatic masses in the seminar ampullae).

Species	Sample	Length (mm)	Weight (g)	Sex	Maturity	N° AD
*Parapenaues longirostris*	8	27.20	7.60	M	2E	0
	10	27.60	9.10	M	2B	0
	14	26.40	9.90	M	2B	0
	16	29.00	8.40	M	2E	0
	19	26.00	6.70	M	2E	0
	26	27.20	9.70	F	2B	0
	32	26.20	7.40	F	2B	0
	34	25.50	7.80	F	2B	0
	35	25.00	7.40	F	2B	0
	36	26.00	4.70	F	2B	0
	38	26.80	8.70	F	2B	0
	41	27.10	9.20	F	2B	0
	44	26.00	8.40	F	2B	0
*Aristeus antennatus*	51	49.60	36.50	F	2E	0
	53	51.50	41.70	F	2E	0
	56	50.30	40.80	F	2E	0
	60	37.50	19.00	F	2E	0
	61	42.50	25.10	F	2E	0
	76	50.80	41.50	F	2E	0
	77	49.30	38.80	F	2E	0
	78	41.90	23.70	F	2B	0
	80	43.00	30.70	F	2B	0
	86	50.50	34.30	F	2B	0
	87	55.50	45.90	F	2B	0
	89	48.10	33.60	F	2B	0
	93	56.00	45.90	F	2B	0
	94	52.00	41.60	F	2B	0
	98	46.80	37.40	F	2B	0
*Aristaeomorpha foliacea*	105	36.00	17.11	F	2E	0
	107	40.0	19.64	F	2E	0
	109	34.80	11.10	F	2E	0
	122	37.50	18.99	F	2E	0
	125	52.00	42.23	F	2E	0
	132	31.00	9.00	F	2E	0

**Table 3:** Size classes (mm) and number of the MPs isolated from the species analyzed this study.

Size Classes	Size Range	*P. longirostris*	*A. antennatus*	*A. foliacea*
I	0.10–0.49	12	11	24
II	0.50–0.99	15	16	8
III	1.00–1.49	11	14	10
IV	1.50–1.99	13	12	10
V	2.00–2.49	12	7	4
VI	2.50–2.99	7	7	5
VII	3.00–3.49	4	3	4
VIII	3.50–3.99	1	4	2
IX	4.00–4.99	1	3	1
X	≥5.00	7	1	1

## Data Availability

The data presented in this study are available within the article.
